# Extracorporeal Shockwave Lithotripsy for Cystine Stones in Children: An Observational, Retrospective, Single-Center Analysis

**DOI:** 10.3389/fped.2021.763317

**Published:** 2021-11-16

**Authors:** Nicolas Vinit, Antoine Khoury, Pauline Lopez, Laurence Heidet, Nathalie Botto, Olivier Traxer, Olivia Boyer, Thomas Blanc, Henri B. Lottmann

**Affiliations:** ^1^Department of Pediatric Surgery and Urology, Necker-Enfants Malades Hospital, APHP, Paris, France; ^2^Department of Pediatric Nephrology, Reference Center for Inherited Renal Disease (MARHEA), Necker-Enfants Malades Hospital, APHP, Paris, France; ^3^INSERM UMR 1163, Laboratory of Inherited Kidney Diseases, Imagine Institute, Université de Paris, Paris, France; ^4^Department of Urology, Tenon Hospital, APHP, Paris, France; ^5^Groupe de Recherche Clinique sur la Lithiase Urinaire (GRC no 20), Tenon Hospital, Sorbonne Université, Paris, France; ^6^Sorbonne Université, Paris, France; ^7^Université de Paris, Paris, France; ^8^INSERM U1151-CNRS UMR 8253, Université de Paris, Paris, France

**Keywords:** cystinuria, cystine stone, extracorporeal shockwave lithotripsy, pediatric nephrolithiasis, stone free

## Abstract

**Purpose:** Cystinuria is a genetic disorder characterized by a defective reabsorption of cystine and dibasic amino acids leading to development of urinary tract calculi from childhood onward. Cystine lithiasis is known to be resistant to fragmentation. The aim was to evaluate our long-term experience with extracorporeal shockwave lithotripsy (ESWL) used as first-line urological treatment to treat cystine stones in children.

**Methods:** We retrospectively reviewed the charts of all children who underwent ESWL for cystine stone. We assessed the 3-month stone-free rate, according to age, younger (group 1) or older (group 2) than 2 years old.

**Results:** Between 2003 and 2016, 15 patients with a median (IQR) age at first treatment of 48 (15–108) months underwent ESWL in monotherapy. Median age was, respectively, 15 and 108 months in each group. The median (IQR) stone burden was 2,620 (1,202–8,265) mm^3^ in group I and 4,588 (2,039–5,427) mm^3^ in group II (*p* = 0.96). Eleven patients had bilateral calculi. ESWL was repeated on average 2.4 times, with a maximum of 4 for patients of group I, and 4.8 times, with a maximum of 9 for group II (*p* > 0.05). ESWL in monotherapy was significantly more efficient to reach stone-free status for children under 2 years of age: 83% vs. 6.2% (*p* = 0.040). The median (IQR) follow-up of the study was 69 (42–111) months.

**Conclusion:** ESWL appears as a valid urological option for the treatment of cystine stones, in young children. Even if cystine stones are known to be resistant to fragmentation, we report 83% of stone-free status at 3 months with ESWL used in monotherapy in children under 2 years old with cystinuria. In older children, the success rate is too low to recommend ESWL as a first line approach.

## Introduction

Cystinuria is a rare autosomal recessive genetic disorder characterized by defective reabsorption of cystine and other dibasic amino acids (ornithine, lysine, and arginine) in the renal proximal tubule and intestinal epithelial cells (1). Dysfunction of this transport mechanism is due to mutations in solute carrier genes *SLC3A1* or in *SLC7A9* (2). Cystinuria is responsible for 5 to 10% of pediatric stones (3) and more than half of patients will develop stones throughout their lifetime, with a higher risk of recurrence, bilaterality (4), and loss of renal function (5–7) than children with non-cystine stones. Although first symptoms often occur during childhood (3), cystinuria can be suspected prenatally, warranting early medical treatment (8–10).

In addition to mandatory medical management, pediatric upper urinary tract stones can be treated using different modalities according to patient's age, stone localization/size, and surgeon's experience: extracorporeal shockwave lithotripsy (ESWL), percutaneous nephrolithotomy (PCNL), retrograde rigid or flexible ureteroscopy (RUS), or open nephrolithotomy (ON) (11). The goal is to reach a stone-free status to avoid recurrence.

Previous reports have demonstrated that ESWL was effective and safe in infants and young children (12, 13) while other techniques (PCNL and RUS) may be more challenging and associated with significant complications, especially in the prepubertal period (14, 15). Cystine stones are known to be particularly resistant to intra- or extracorporeal fragmentation (11) and no consensus has been reached regarding the preferred and age-appropriate technique.

This study's aim was to assess the efficacy of ESWL in treating cystine stones in children according to the age of treatment.

## Materials and Methods

With the approval from the Necker-Enfants Malades Hospital's institutional review board, we retrospectively reviewed the data of all children (younger than 16 years old) treated in our center for cystine stones with at least one urological procedure for upper urinary tract stones. We selected those with a diagnosis of cystinuria and at least once treated using ESWL. All patients were diagnosed with cystinuria based on urine analysis (Brandt reaction and/or crystalluria study with infrared spectrophotometry) prior to any urologic or medical treatment. There were no exclusion criteria.

Data collected included age, gender, familial history of cystinuria, parental consanguinity, circumstance of diagnosis, stones' location, medical management, age at first urological treatment, stone burden (mm^3^), type of procedure (ESWL, PCNL, ON, or RUS), and number of procedures to reach a stone-free status.

Preoperative evaluation of stone diameter was done using kidney–ureter–bladder (KUB) x-ray, ultrasonography (US), and/or computed tomography (CT) scan for right and left upper urinary tract. A spherical shape of the stones was assumed to determine the stone burden for each child, using measurements in coronal, sagittal, and axial planes on KUB x-ray, ultrasound, and/or CT scan.

ESWL procedures were performed as first line/option in the initial surgical management for most patients or as an adjunct after prior urological procedure in some. Sessions were conducted under general anesthesia by the same pediatric urologist (HL), using a Dornier® Compact Delta lithotripter. Ultrasound was mainly used as a targeting system for renal stones. Ultrasound and/or fluoroscopy was used to target ureteral stones. Power was set on 4 for renal stones and 5 for ureteral stones. A maximum of 2,500 shocks on the kidney and 3,500 on the ureter were delivered per site and per session. Stone fragments were analyzed in all patients using a Fourier-transform infrared spectrometer.

Patients were divided into two groups according to age at treatment: ≤ 2 years old (group 1) and >2 years old (group 2). Results were analyzed separately for each group.

Follow-up included KUB and ultrasound at 1 month following urologic procedure for all patients in addition to the nephrologic follow-up. In case of residual fragments, repeat KUB and ultrasound were performed at 3 months. If patients were stone-free, KUB–ultrasound were performed at 6 months and then on a yearly basis. ESWL was considered a monotherapy if no other urological procedure was necessary to achieve a stone-free status per treated site. Success of treatment (stone-free status) was defined for each treated side (right and/or left) as no visible remaining stones on KUB and US, or CT scan 3 months after the end of initial urological treatment. Recurrence was defined as a newly appeared stone during the follow-up period.

The study was conducted in accordance with French legislation, Good Clinical Practices, and the Declaration of Helsinki.

Data were expressed as mean (range) for continuous variables, and as numbers and percentages for categorical variables. Mann–Whitney test or Student's *t*-test was used to compare continuous variables according to normal distribution (Shapiro–Wilk's test) and chi-square or Fischer's exact test for categorical variables. A *p* < 0.05 was considered significant.

## Results

Between 2003 and 2016, 210 children were treated in our department for upper urinary tract stones. Eighteen children (7%) were diagnosed with cystine stones. Three of them were never treated with ESWL and were therefore excluded from further analysis.

Seven children were younger than 2 years old (group 1) and eight were older than 2 years old (group 2). There were eight boys and seven girls.

Parental consanguinity was noted in three cases (20%) and a family history of cystine stones in six cases (40%) (Table 1). In two cases (13%), cystinuria was suspected prenatally based on a hyperechoic colon during the third trimester of pregnancy. Symptoms included urinary tract infection (*n* = 5; 33%), renal colic (*n* = 4; 31%), anuria (*n* = 1; 7%), and stone fragments in the diaper (*n* = 1; 7%). One child was diagnosed through sibling screening of an index case.

**Table 1:** Patients' characteristic.

						**Presentation**						**Medical treatment**	
**No**.	**Group**	**Age at first urological procedure**	**Gender**	**Parental consanguinity**	**Familial history of cystine**	**Antenatal diagnosis**	**UTI**	**Screening**	**ANU**	**RC**	**Sand in diaper**	**Hyperhydration + urine alkalization**	**Sulfhydryl compounds**
1	1	18 mo	Male	–	–	–	+	–	–	–	–	+	+
2	1	23 mo	Male	–	–	–	+	–	–	–	–	+	+
3	1	12 mo	Male	–	+	–	–	–	+	–	–	+	+
4	1	19 mo	Female	–	–	+	–	–	–	–	–	+	–
5	1	14 mo	Female	–	+	–	+	–	–	–	–	+	+
6	1	15 mo	Male	–	–	–	–	–	–	–	+	+	–
7	1	15 mo	Female	–	–	–	–	–	–	+	–	+	–
8	2	3 yo	Male	+	+	+	–	–	–	–	–	+	+
9	2	5 yo	Female	–	–	–	+	–	–	–	–	+	+
10	2	6 yo	Male	–	–	–	–	–	–	+	–	+	+
11	2	9 yo	Male	–	+	–	–	+	–	–	–	+	+
12	2	13 yo	Female	+	–	–	+	–	–	–	–	+	+
13	2	13 yo	Female	–	+	–	–	–	–	–	–	+	+
14	2	9 yo	Male	+	+	–	–	–	–	+	–	+	+
15	2	11 yo	Female	–	–	–	–	–	–	+	–	+	+
**Total**				3	6	2	5	1	1	4	1	15	12

*UTI, urinary tract infection; ANU, anuria; RC, renal colic; Mo, months old; Yo, years old*.

All patients received medical management: hyperhydration and urine alkalization. Most of them received sulfhydryl compounds (*n* = 12; 80%) at some point.

Cystine stones were bilateral in six children in group 1 (86%) and five children in group 2 (62%) (Tables 2, 3). The mean stone burden was 2,533 (171–9,202) mm^3^ in group 1 and 5,791 (696–17,157) mm^3^ in group 2 (*p* = 0.02). The first urological procedure was performed at a mean age of 17 (12–23) months in group 1 and 8.6 (3–13) years in group 2.

**Table 2:** Results in group 1 (children younger than 2 years old).

			**Stone location at urological management**				**Urological management in order to reach a stone–free status**					**Outcome after initial management**				
**#**	**Bilateral stones at diagnostic**	**Side**	**Kidney (calyceal, pelvis)**	**Ureter**	**Bladder**	**Total stone burden before first procedure (mm^**3**^)**	**ESWL**	**PCNL**	**ON**	**RUS**	**Other**		**Recurrence**	**Time to initial recurrence (months)**	**Stone–free status at the end of follow–up**	**Follow-up (months)**
1	**Yes**	R	+	–	–	382	1	0	0	0	0	SF	No	–	**Yes**	84
		L	+	–		171	2	0	0	0	0	**RF**	No	–		
2	**Yes**	R	+	–	–	2,572	1	0	0	0	0	SF	No	–	**Yes**	120
		L	+	–		524	1	0	0	0	0	SF	No	–		
3	**Yes**	R	+	+	–	510	1	0	0	0	0	SF	No	–	**Yes**	60
		L	+	+		601	2	0	0	0	0	SF	No	–		
4	No	R	+	–	–	2,144	1	0	0	0	0	SF	No	–	**Yes**	120
		L	–	–		–	–	–	–	–	–	–	–			
5	**Yes**	R	+	–	–	6,020	1	0	0	0	0	**RF**	No	–	**Yes**	108
		L	–	+		7,239	1	0	0	0	**1**	SF	No	–		
6	**Yes**	R	–	+	+	2,144	0	0	0	0	**1**	SF	No	–	**Yes**	60
		L	+	–		9,202	1	0	0	0	0	SF	No	–		
7	**Yes**	R	+	–	–	524	1	0	0	0	0	SF	No	–	**Yes**	36
		L	+	–		905	1	0	0	0	0	SF	No	–		
																
																
**Total**	6		11	4	1	–	14	0	0	0	2		0		7	

*ESWL, extracorporeal shockwave lithotripsy; PCNL, percutaneous nephrolithotomy; ON, open nephrolithotomy; RUS, retrograde ureteroscopy; SF, stone free; RF, residual fragment; R, right; L, left*.

**Table 3:** Results in group 2 (children older than 2 years old).

			**Stone location at urological management**				**Urological management in order to reach a stone–free status**					**Outcome after initial management**				
**#**	**Bilateral stones at diagnostic**	**Side**	**Kidney (calyceal, pelvis)**	**Ureter**	**Bladder**	**Total stone burden before first procedure (mm^**3**^)**	**ESWL**	**PCNL**	**ON**	**RUS**	**Other**		**Recurrence**	**Time to initial recurrence (months)**	**Stone–free status at the end of follow–up**	**Follow-up (months)**
8	No	R	+	–	–	14,137	5	0	0	0	0	SF	**No**	–	**Yes**	60
		L	–	–		–	–	–	–	–	–	**–**	**–**			
9	No	R	+	–	–	7,714	3	1	0	0	0	**RF**	**Yes**	26	No	70
		L	–	–		–	–	–	–	–	–	**–**	**–**	–		
10	**Yes**	R	+		–	3,984	3	0	1	0	0	SF	**Yes**	72	No	140
		L	+	–		4,188	5	0	0	1	0	SF	**Yes**	72		
11	No	R	+	–	–	17,157	6	0	0	3	0	SF	No	–	**Yes**	60
		L	–	–		–	–	–	–	–	–	–	–	–		
12	**Yes**	R	+	–	–	4,180	2	0	1	0	0	**RF**	**Yes**	12	No	92
		L	+	–		696	1	0	0	1	0	**RF**	**Yes**	24		
13	**Yes**	R	+	–	–	5,362	1	0	1	0	0	SF	No	–	**No**	156
		L	+	–		4,200	0	0	1	0	0	SF	**Yes**	72		
14	**Yes**	R	+	–	–	4,080	0	0	1	0	0	SF	Yes	50	**No**	74
		L	+	–		4,220	1	0	1	4	0	SF	**Yes**	62		
15	**Yes**	R	+	–	–	4,000	1	0	0	0	0	**RF**	**Yes**	24	No	90
		L	+	–		1,372	2	0	0	0	0	SF	**Yes**	24		
**Total**	**5/8**		12	0	0		30	1	6	9	0		10		2	

*ESWL, extracorporeal shockwave lithotripsy; PCNL, percutaneous nephrolithotomy; ON, open nephrolithotomy; RUS, retrograde ureteroscopy; R, right; L, left*.

A total of 44 ESWL session were performed, 14 in group 1 and 30 in group 2. Overall treatment outcomes are summarized in Tables 2, 3.

In group 1, ESWL was used as a monotherapy to treat at least one side for all children and allowed to reach a stone-free status in all treated cases but two (85%).

In one case, ESWL was followed by a ureteral steinstrasse requiring an ureterotomy to remove an obstructive residual fragment (RF) after RUS failure (case # 5).

During a single general anesthesia, patient #6, from abroad, had ESWL on the left side (caliceal stone) and a ureterotomy and cystotomy to remove a bladder and a large right lower ureteric stone.

The two children (#1 and #5) with RF mobilized their stone after 3 years of medical treatment and required cystotomy and ureteroscopy, respectively.

In group 2, ESWL was successful as a monotherapy to reach a stone-free status in one side in only two patients (#8 and #15). In other sides, either the child had small RF after ESWL or at least one other kind of urological procedure was necessary following ESWL.

In case # 8, ESWL was followed by acute urinary retention because of a fragment obstructing the urethral meatus and was extracted under sedation using small forceps.

ESWL was repeated on an average 1.1 times per side, with a maximum of 2 per side for group 1 patients and an average of 2.7 times per side, with a maximum of 6 for group 2 (*p* = 0.04).

ESWL used as a monotherapy was significantly more efficient to reach stone-free status per treated side without recurrence in children younger than 2 years old: 85% (*n* = 11/13) in group 1 against 8% (*n* = 1/13) in group 2 (*p* = 0.002).

The median follow-up was 84 (36–156) months. No patient was lost to follow up.

At last follow-up, no recurrence occurred and all children were stone free in group 1. All children with RF recurred and only two children were stone free in group 2.

## Discussion

Cystinuria is a rare inherited disorder whose first clinical signs can appear during early childhood (3). Treatment is challenging. Children with cystinuria are known to be at higher risk of early surgical management than those with non-cystine stones (16). Prevention of stone formation through medical management is the first line and one of the most important part (17). The aim of the medical management is to decrease urinary cystine concentration using dietary measures and to increase crystals' solubility. Prophylactic measures include proper and permanent hydration with fluid intake (at least 3 L/1.73 m^2^/day) and urine alkalinization (18, 19). D-Penicillamine or Tiopronine (sulfhydryl compounds) may be added to increase the solubility of cystine up to 50 times (3). However, compliance is difficult to obtain in young patients and side effects may lead to treatment interruption (20, 21).

Cystinuria may be suspected prenatally when an hyperechogenic colon is observed at third-trimester US (8–10). In our study, two patients had a prenatal diagnosis of cystinuria but early medical management could not avoid cystine stone formation. Most patients will require repeat urological procedures during childhood despite proper preventive measures and medical treatment (6, 22). The mean age at first ESWL treatment was 5.2 years in our study. Half of patients received their first procedure before 2 years of age (group 1).

Urological management for cystine stones is challenging. Some authors suggest that ESWL should be restricted to stones smaller than 15 to 25 mm (longest diameter) and prefer PCNL or ON for larger stones (3, 23–25). RUS is preferred for distal ureteral stones (26, 27) and represent a suitable alternative when treating fragments refractory to prior ESWL (3, 14, 28) but remains difficult in young patients. Although cystine stones have always been considered as rather resistant to fragmentation (1), reasonable outcomes have been reported in literature on lithotripters with higher power (29–31). Efficacy and safety of ESWL in the treatment of upper urinary tract stones in children have already been established (12, 23, 32–34). In our study, a total of 62 (16 in group 1, 46 in group 2) urological procedures were performed on 15 patients, and most of them (71%, *n* = 44/62) were ESWL with an average of three ESWL procedures per child. ESWL was used as a monotherapy to treat at least one side for all patients younger than 2 years old and allowed to reach a stone-free status without recurrence in all cases but one. For children older than 2 years old, monotherapy of ESWL allowed to reach the stone-free status without recurrence in only one treated side. ESWL is a low invasive technique that can be used safely in infants. It requires general anesthesia in children but can be repeated several times for the same patient. The two complications observed were a ureteral steinstrasse requiring an open procedure to remove ureteral stones and an acute urinary retention easily managed without general anesthesia. Moore et al. have recently highlighted the importance to reach a stone-free status after the initial management. According to their results, stone-free patients after their first treatment had lower recurrence rate and fewer procedures during their follow-up (35). Our excellent results of ESWL monotherapy in treating cystine stones in young children (<2 years old) (85% of stone free status) suggest that it could be the reference technique for this age group even for large cystine stones, when combined with proper medical management. Moreover, shockwave transmission is probably better in young children and the ureter is also more compliant to the passage of stone fragments (12). The success of cystine stone fragmentation in younger children may be explained by the greater water content in their tissue and their shorter shockwave transmission path, the fact that the procedure was carried out under general anesthesia, thus minimizing shockwave imprecision due to breathing movements, the lower stone burden as observed in our cohort, the better compliance to medical treatment in young children, or possibly the younger age of the cystine stones (12, 36–38). Conversely, we showed that the older the child is, the poorer the efficacy of ESWL is to reach a stone-free status, with a statistically significative difference. The youngest patient in group 2, aged 3 years, required five ESWL sessions to reach a stone-free status. These findings may be related to a heavier initial stone burden in children aged >2 years old. A lack of observance to medical treatment for school children and adolescents may also contribute to these results, but our study did not address this question.

The advancements in miniaturization of ureteroscopes allow to perform ultra-minimally invasive procedures in children (39–43). These technologies represent new alternative modalities of treatment to ESWL in cystinuria for children but remains challenging, especially for the youngest. A recent systematic review of ureteroscopy for stone disease in the pediatric population highlighted a higher failure and complication rate of RUS in young children (14). Patient's age has been proven to be a strong significant predictor of failure for YAG laser ureteroscopic lithotripsy (44). Considering, on one hand, our good results of ESWL for the treatment of cystine stones for children younger than 2 years old and the higher complication rate of RUS in this population, on the other hand, we suggest an age-dependent urological strategy: a reasonable option is to treat young children with ESWL, particularly infants, whereas RUS and PCNL seem to be more appropriate and accessible to older children.

Although short, our series constitute to our knowledge the largest published pediatric series of nephro-urological management of cystine stones with ESWL, which was the only option besides open surgery for our earliest patients, with a follow-up up to 156 months. Analyzed data did not include the exact caliceal location of the stones, which may have limited our results since location of the stones are potential predictor ESWL success.

Cystinuria remains a rare disease, explaining the limited number of patients and the large study period. Further prospective and multicenter studies with a large number of patients are required to determine adequate urological treatment strategies in cystine stones in children.

## Conclusion

ESWL appears to be a valid urological option for the treatment of cystine stones, in infants and toddlers. Even if cystine stones are known to be resistant to fragmentation, we report excellent results achieved with ESWL used as a monotherapy in children younger than 2 years old. For older patients, results achieved with ESWL as a first-line approach are poorer and another treatment strategy should be preferred.

## Data Availability Statement

The raw data supporting the conclusions of this article will be made available by the authors, without undue reservation.

## Ethics Statement

The studies involving human participants were reviewed and approved by Necker-Enfants Malades Institutional Review Board. Written informed consent from the participants' legal guardian/next of kin was not required to participate in this study in accordance with the national legislation and the institutional requirements.

## Author Contributions

NV: data analysis, manuscript drafting, and manuscript revision. AK and PL: data collection, data analysis, and manuscript drafting. LH, NB, OT, and OB: critical revision and manuscript review. TB: data collection, data analysis, critical revision, and manuscript drafting. HL: study design, manuscript drafting, critical revision, and manuscript review. All authors contributed to the study conception and design and read and approved the final manuscript.

## Conflict of Interest

The authors declare that the research was conducted in the absence of any commercial or financial relationships that could be construed as a potential conflict of interest.

## Publisher's Note

All claims expressed in this article are solely those of the authors and do not necessarily represent those of their affiliated organizations, or those of the publisher, the editors and the reviewers. Any product that may be evaluated in this article, or claim that may be made by its manufacturer, is not guaranteed or endorsed by the publisher.

## Abbreviations

ESWL: Extracorporeal shockwave lithotripsy
PCNL: Percutaneous nephrolithotomy
RUS: Retrograde ureteroscopy
ON: Open nephrolithotomy
KUB: Kidney–ureter–bladder
US: Ultrasonography
CT: Computed tomography
RF: Residual fragments.
